# Planned Impella 5.5 Support via Brachiocephalic Artery or Ascending Aorta for Low Ejection Fraction, High-Risk Cardiac Surgery

**DOI:** 10.3390/jcdd13100475

**Published:** 2026-09-22

**Authors:** Andreas Habertheuer, Anjeli Reyes Macaranas, Sameer Hirji, Pimchanok Junnil, Amy E. Hackmann, Margaret R. Connolly, Thoralf M. Sundt, Akinobu Itoh

**Affiliations:** Division of Cardiac Surgery, Brigham and Women’s Hospital, Mass General Brigham, Harvard Medical School, Boston, MA 02115, USA; ahabertheuer@bwh.harvard.edu (A.H.);

**Keywords:** high-risk cardiac surgery, low ejection fraction (EF), Impella 5.5, temporary mechanical circulatory support (MCS)

## Abstract

*Background:* Surgically implanted microaxial flow pumps are increasingly being utilized to treat cardiogenic shock; however, data on their use in the realm of cardiac surgery are scarce. We report our single-institution experience in low-ejection-fraction, high-risk patients with a planned weaning strategy from cardiopulmonary bypass directly to Impella 5.5 inserted via the brachiocephalic artery or ascending aorta following corrective cardiac surgery. *Methods*: A prospectively maintained institutional database was queried from 2022 through to 2025. Primary endpoints included feasibility of implantation, weaning rate, and bedside removal; secondary endpoints included freedom from all-cause mortality at discharge and 1 year, in-hospital thromboembolic complications, postoperative pharmacologic hemodynamic support using vasoactive inotropic score (VIS), de novo renal failure, and revision for bleeding. Descriptive statistics were applied. *Results:* Sixteen patients (median age 66.5 years [IQR; interquartile range, 52.5–70.0], STS score 12.1% [IQR, 5.3–14.2%] and EF 20.0% [IQR, 15.0–31.5%]) underwent coronary revascularization (*n* = 6), valve procedures (*n* = 4) or a combination thereof (*n* = 6) and were weaned from cardiopulmonary bypass to Impella 5.5 implanted via the ascending aorta (25%) or brachiocephalic artery (75%). Primary endpoints were met in 100% (feasibility of implantation), 94% (weaning rate), and 100% (bedside removal). In terms of secondary endpoints, freedom from mortality at discharge and one year was 94.8%; there were two non-disabling strokes (12.6%) while still on Impella 5.5 support; the median VIS at the time of ICU arrival was 12.8 (IQR 10.8–22.1); renal failure occurred in two patients and four patients required revision for bleeding. *Conclusions:* Impella 5.5-supported cardiac surgery appears safe and may open a new avenue for high-risk patients.

## 1. Introduction

Patients undergoing high-risk cardiac surgery, especially those with severely impaired left ventricular (LV) ejection fraction (EF) may face a myriad of postoperative complications leading to increased morbidity and mortality. This patient population is especially prone to post-cardiotomy shock, where reduced cardiac output along with increased left ventricular end-diastolic pressure (LVEDP) and volume (LVEDV), increased LV wall tension, and impaired coronary perfusion create a physiologic environment that is non-conducive to cardiac recovery. Various temporary mechanical circulatory support (tMCS) platforms exist to ameliorate downstream effects, including intra-aortic balloon pump (IABP) and veno-arterial extracorporeal membrane oxygenation (V-A ECMO) with varying effects on cardiac output, afterload, and coronary perfusion. IABP increases myocardial perfusion while decreasing afterload and myocardial oxygen consumption; however, augmentation of cardiac output and left ventricular unloading is minimal. ECMO, on the other hand—while capable of delivering full cardiac output—increases left ventricular afterload and, in many instances, does not effectively unload the LV without further adjuncts.

Left ventricular unloading is increasingly recognized as an ideal management strategy in this scenario by providing full cardiac output support while venting the left ventricle and potentially allowing for cardiac recovery. As such, the Impella family of microaxial flow devices (Abiomed under Johnson and Johnson, Danvers, MA, USA) have emerged as pivotal tools in the treatment of cardiogenic shock. The Impella 5.5 is now recognized as a primary therapy of choice for myocardial infarction (MI)-related cardiogenic shock jointly by the American Association for Thoracic Surgery, the Society of Thoracic Surgeons, and the European Association of Cardiothoracic Surgery [1]. This trend has also extended to the cardiac surgery community [2,3,4,5,6,7], although there are no universally accepted guidelines.

The full-support version (Impella 5.5) consists of a 19Fr motor housing and a 21Fr rigid cannula that propels blood from the left ventricle across the aortic valve into the ascending aorta with a displacement volume of up 5.5 L per minute. There are two primary scenarios for the use of Impella 5.5: (1) bridging acutely ill patients with cardiogenic shock to recovery or definitive therapy (corrective surgery, durable MCS or heart transplant) and (2) providing hemodynamic support postoperatively as an escalation strategy to facilitate cardiac recovery. In the latter scenario, patients are typically weaned from cardiopulmonary bypass (CPB) and—after failing lesser degrees of support such as pharmacologic therapy, IABP, and/or ECMO—are escalated to tMCS as a rescue attempt.

Herein, we report our experience with a planned, “Impella first” strategy for immediate left ventricular unloading in high-risk patients with severely reduced LVEF weaned directly from CPB to Impella 5.5 following coronary revascularization and/or valve procedures.

## 2. Materials and Methods

### 2.1. Study Design and Patient Selection

This study was approved by the Mass General Brigham Institutional Review Board with waived consent (approval number: 2025P000971, approval date 18 April 2025). A prospectively maintained institutional database was queried from 2022 to 2025. Patients with coronary artery disease and EF <20% and/or severe aortic valve or mitral valve insufficiency with EF <25% were included (Figure 1). All patients underwent preoperative work-up via echocardiography, coronary angiography, and computed tomography (CT) of the chest. Primary endpoints included feasibility of implantation via the brachiocephalic artery or ascending aorta, weaning rate to explantation, and feasibility of bedside removal; secondary endpoints included freedom from all-cause mortality at discharge and 1 year, in-hospital thromboembolic complications, postoperative pharmacologic hemodynamic support using vasoactive inotropic score (VIS), de novo renal failure, and revision for bleeding. VIS was calculated as a weighted sum of all administered inotropes and vasoconstrictors using the following formula [7]: 100 × Epinephrine dose (μg/kg/min) + 100 × Norepinephrine dose (μg/kg/min) + 10,000 × Vasopressin dose (U/kg/min). All patients had at least two transthoracic echocardiography (TTE) assessments: during the Impella weaning phase and prior to discharge. Descriptive statistics were applied.

### 2.2. Implant Technique

In all patients, a straight 8 or 10 mm Dacron graft was sewn to the brachiocephalic artery or the ascending aorta at the beginning of each planned cardiac operation (coronary revascularization, valve procedures or a combination thereof) using a partial clamp and tunneled to the right supraclavicular region via a counter incision to allow for chest closure and subsequent bedside device removal. Graft location (brachiocephalic artery versus ascending aorta) was chosen to maintain a minimal distance between the level of the aortic valve and graft anastomosis of 7 cm, per manufacturer recommendations. A guidewire was advanced to the level of the aortic root under transesophageal echocardiography (TEE) guidance before initiation of cardiopulmonary bypass (CPB), avoiding fluoroscopy. All planned valvular and/or coronary procedures were then performed on CPB with the cross-clamp over the guidewire in the ascending aorta. Prior to removal of the aortic cross-clamp, the wire was guided into the LV cavity under direct vision via a small aortotomy (CABG or mitral valve replacements). Aortic valve replacements were done in the conventional way; then, the guidewire was passed through the tissue valve prior to closure of the aortotomy. Following removal of the cross-clamp, Impella 5.5 was advanced into the LV under TEE guidance without fluoroscopy. The Impella support was initiated and incrementally increased and optimized as the CPB was weaned with minimal pharmacologic support. All patients were managed postoperatively according to best practice critical care guidelines.

### 2.3. Postoperative Anticoagulation Management

As blood is in direct contact with the Impella surfaces, anticoagulation is required to minimize risk of thromboembolic complications. While sodium bicarbonate is the preferred and FDA-approved purge solution, unfractionated heparin remains the preferred systemic anticoagulation. In this study, while on Impella support, all patients were maintained on a therapeutic heparin drip, as guided by partial thromboplastic time (PTT) or anti Xa levels, per institutional guidelines.

### 2.4. Data Management and Statistical Analysis

Continuous variables are presented as mean ± standard deviation when normally distributed and as median interquartile range (IR) in cases of non-normality. Categorical variables are presented as frequencies. All data are 100% complete and there are no missing values. Unadjusted survival outcomes are displayed using Kaplan–Meier curves (Figure 2). All statistical analyses were performed using R Version 4.5.1 “Great Square Root” (Vienna, Austria). The following R packages were used for analysis: “ggplot2”, “ezfun”, “dplyr” and “survival”.

## 3. Results

### 3.1. Demographics and Preoperative Parameters

From March 2022 through June 2025, 16 consecutive high-risk, low-EF patients undergoing cardiac surgery who underwent a planned weaning approach from cardiopulmonary bypass (CBP) directly to Impella 5.5 implanted via the brachiocephalic artery or ascending were identified at a single institution. Baseline, operative, and follow-up data were 100% complete. The preoperative demographics are summarized in Table 1. The median age was 65.5 years (interquartile range [IQR] 52.5–70.0 years), median BMI was 24.8 (IQR, 22.2–29.7), and 93.8% of the patients were male. Comorbid conditions included hypertension and CAD (both 56.3%), hyperlipidemia (50%), nicotine abuse (43.8%), atrial fibrillation (25.0%), diabetes, renal failure, and COPD (all 18.8%). In terms of preoperative baseline cardiac function, the median EF across all patients was 20.0% (IQR, 15.0–31.5).

### 3.2. Primary Endpoints

#### 3.2.1. Feasibility of Implantation

The intraoperative details are summarized in Table 2. Cardiac surgical procedures included isolated coronary artery revascularization (*n* = 6, 37.5%), valvular procedures (*n* = 4, 25%), and valvular procedures combined with coronary artery revascularization (*n* = 6, 37.5%). Of all devices, 12 were inserted via the brachiocephalic artery (75%) and 4 were inserted via the ascending aorta (25%). All patients were weaned from cardiopulmonary bypass (CPB) directly to Impella 5.5 without the need for additional mechanical circulatory support. As described above, Impella 5.5 flows were optimized for every patient individually, balancing LV decompression and suction events; the median initial Impella flows were 3.0 L/min (IQR, 3–3.7). Of note, two patients were inadequately supported on Impella 5.5 secondary to biventricular failure and required treatment escalation to another mechanical circulatory support system within the first postoperative night; one patient received a percutaneous temporary RVAD (Impella RP Flex, removed on postoperative day 8 on continued Impella 5.5 support) and one patient was cannulated onto peripheral VA-ECMO (decannulated on postoperative day 10 on continued Impella 5.5 support).

#### 3.2.2. Impella 5.5 Weaning Rates and Bedside Removal

The postoperative details are presented in Table 3. The median duration of Impella 5.5 support was 8.5 days (IQR 6.5–13). Inotropic and vasopressor hemodynamic support was weaned over 13 days (IQR, 7–16). Postoperative cardiac function measured by echocardiography stabilized, as evidenced by an ejection fraction of 20.0% (IQR, 15.0–31.5), 27.5% (IQR, 25.0–30.5), and 35% (IQR, 23–35) at baseline, postoperative day 4 (IQR, 3.5–5.5), and postoperative day 9 (IQR, 7–16). For all patients combined, the first recorded cardiac index (CI) following Impella 5.5 removal was 3.0 L/min/m^2^ (IQR 2.7–3.5). Following successful Impella weaning, all devices were removed at bedside with no bleeding or thromboembolic complications. Of note, one patient with extensive ischemic cardiomyopathy and a baseline EF of 15% ultimately failed to wean off Impella support and received a durable left ventricular assist device (LVAD) on postoperative day 28. The Median postoperative ICU and hospital length of stay were 18.5 days (IQR, 11.0–32.5) and 26 days (IQR,17.0–63.5), respectively.

### 3.3. Secondary Endpoints

#### 3.3.1. Mortality and Morbidity Outcomes

The median follow-up for the entire cohort was 10 months (Figure 2, IQR, 7.5–20.5). For the entire cohort, the STS predicted risk of operative mortality was 12.1% (5.3–14.2). The observed freedom from all-cause mortality at 1 year was 94.8% (one operative mortality on postoperative day 27). Of note, we observed two non-disabling strokes (12.6%) without residual deficits at time of hospital discharge while still on Impella 5.5 support, both in the setting of extensive intra-cranial and extra-cranial atherosclerotic disease. Not a single case of mesenteric or extremity malperfusion was observed.

At time of ICU arrival, patients were on low to moderate doses of inotropes and vasopressors; the median vasoactive inotropic score was 13.8 (IQR, 10.8–27.9). During hospitalization, de novo renal failure occurred in two patients and four patients required revision for bleeding.

#### 3.3.2. Outcomes Beyond Primary and Secondary Endpoints

Outcomes beyond the reported primary and secondary endpoints are reported in Table 3.

## 4. Discussion

Temporary mechanical circulatory support (tMCS) is now the standard of care for managing acute cardiogenic shock. Impella-facilitated tMCS is especially relevant given its ability to unload the LV while providing full cardiac output support, effectively breaking the cycle of high LVEDP and LVEDV leading to LV dilation, high LV wall tension, and impaired coronary perfusion. We sought to evaluate an “Impella first” paradigm, where patients with low EF undergoing coronary revascularization and/or valvular procedures (aortic valve replacements, mitral valve replacements, tricuspid valve repairs) were weaned from cardiopulmonary bypass directly to Impella 5.5 for immediate LV unloading and cardiac output support. Our results indicate that (1) Impella 5.5 can be implanted safely via the supra-aortic brachiocephalic artery or ascending aorta at the time of index cardiac operation; this can be achieved under direct vision and TEE guidance without the use of fluoroscopy enhancing efficacy in the operating room. (2) Tunneling of the graft allows for chest closure and subsequent bedside tMCS removal. (3) Weaning rates are high (in excess of 90%). (4) Morbidity and mortality rates appear acceptable.

### 4.1. Feasibility and Safety of Implantation

Impella 5.5 is typically implanted via a right axillary artery approach in non-cardiac surgery patients [3]. In our experience, we have used the “Impella first” strategy for immediate left ventricular unloading in high-risk patients with severely reduced LVEF undergoing coronary revascularization, valvular procedures (aortic valve replacement, mitral valve replacement, tricuspid valve repair) or a combination thereof. It has to be pointed out that Impella 5.5 cannot be used in conjunction with a mechanical aortic valve and can only be used with extreme caution in conjunction with a mechanical mitral valve. In this series, all valve procedures were conducted with bioprosthetic valves.

For planned post-CPB Impella 5.5 support, we immediately connected an 8 or 10 mm Dacron graft to the brachiocephalic artery or ascending aorta prior to instituting cardiopulmonary bypass. The brachiocephalic artery graft anastomosis for Impella insertion is useful for patients with short ascending aorta, especially patients with smaller body habitus. In addition, this approach appears useful as if we do not spend any additional and unnecessary time on CPB, we avoid an axillary cutdown and the graft is simply tunneled to the right supraclavicular region to allow for bedside access. This allows for easy insertion, stable device position, chest closure, and extubation for early mobilization. Device insertion was feasible in 100% of patients and we did not encounter any technical issues during device insertion such as vascular or valvular injury from the wire or Impella. While four patients had to be re-explored for bleeding, none of the bleeding originated from the Impella graft anastomosis itself. These results are in line with the few available reports [4,5,6]. Eisenga et al. [6] retrospectively reported on 10 patients undergoing Impella 5.5 support in the context of corrective cardiac surgery; however, only 7 patients underwent perioperative device insertion (1 preoperative and 2 postoperative device insertions) and the majority underwent central aortic device placement (60%) while the majority of our patients were accessed via the brachiocephalic artery. Comparable to our experience, the technical success rate was 100%; however, 20% of their patients sustained a cardiac arrest and 50% were re-explored for bleeding.

### 4.2. Impella tMCS Weaning and Bedside Removal

We weaned Impella flows over a couple of days in the ICU under constant monitoring of cardiac output and perfusion markers. Only one patient with extensive ischemic cardiomyopathy and a baseline EF of 15% ultimately failed to wean and received a durable left ventricular assist device (LVAD) on postoperative day 28, suggesting that a high percentage of patients may benefit from this approach. Compared to the series of Eisenga et al. [6], our patients needed longer support (8.5 vs. 5.5 days) for those who were 10 years older (66 vs. 56 years) with a lower baseline EF (20 vs. 25%).

In our experience—and critically for bedside device removal—we tunnel the Dacron graft from the site of vascular access (supra-aortic brachiocephalic artery or ascending aorta) via a counter incision to the right supraclavicular incision. It is important to leave some redundancy and avoid tension on the graft to avoid suture line bleeding of the anastomotic site with the brachiocephalic artery or ascending aorta. The counter incision allows for bedside graft access and control. Following successful Impella weaning, we reopen the counter incision at bedside following application of local anesthesia, encircle the graft with a heavy braided silk suture for proximal control, stop Impella flows, and withdraw the device through the graft. Two steps are critical: (1) the graft should be cut somewhere along the counter incision, and the distal end of the graft should be withdrawn via the Impella skin site to minimize risk of infection, and (2) the proximal end of the graft should be allowed to back-bleed to flush out any potential debris. The graft can then be ligated or closed with a running 4-0 polypropylene sutures.

### 4.3. Morbidity and Mortality

Post-cardiotomy shock has historically been associated with high morbidity as well as mortality rates of more than 50%. In our series, freedom from all-cause mortality at discharge and 1 year was 94.8%, which appears especially remarkable as the STS predicted risk of mortality was 12.1% in this population for operative mortality alone, indicating a lower mortality rate than expected and a potential survival benefit of immediate LV unloading and cardiac output support following cardiotomy. Eisenga et al. [6] reported a survival of discharge as well as 30-day and 90-day survival of 90%, 80%, and 70%, respectively.

### 4.4. Study Limitations

There are limitations to the presented study inherent to the single-institution design. The presented patient cohort is small and lacks a control group. As such, data suggest the feasibility of device implantation, temporary support, and safety of bedside removal; however, the statistical power is too limited to suggest a survival advantage compared to traditional treatment approaches (VA ECMO, IABP, or pharmacologic support). All procedures were performed by a single surgeon at a high-volume institution, and all patients were cared for by a team of anesthesiologists and intensivists with extensive experience in the management of complex cardiac physiologies requiring MCS, limiting generalizability.

## 5. Conclusions

Planned Impella support for high-risk, low-EF cardiac surgery patients is associated with acceptable morbidity and mortality. These patients were weaned from cardiopulmonary bypass directly to Impella 5.5 implanted via the brachiocephalic artery or ascending aorta for immediate LV unloading and augmentation of the cardiac output, as well as a safe and reproducible algorithm for device implantation and bedside removal. Larger-scale studies are desired to provide further answers.

## Figures and Tables

**Figure 1:**
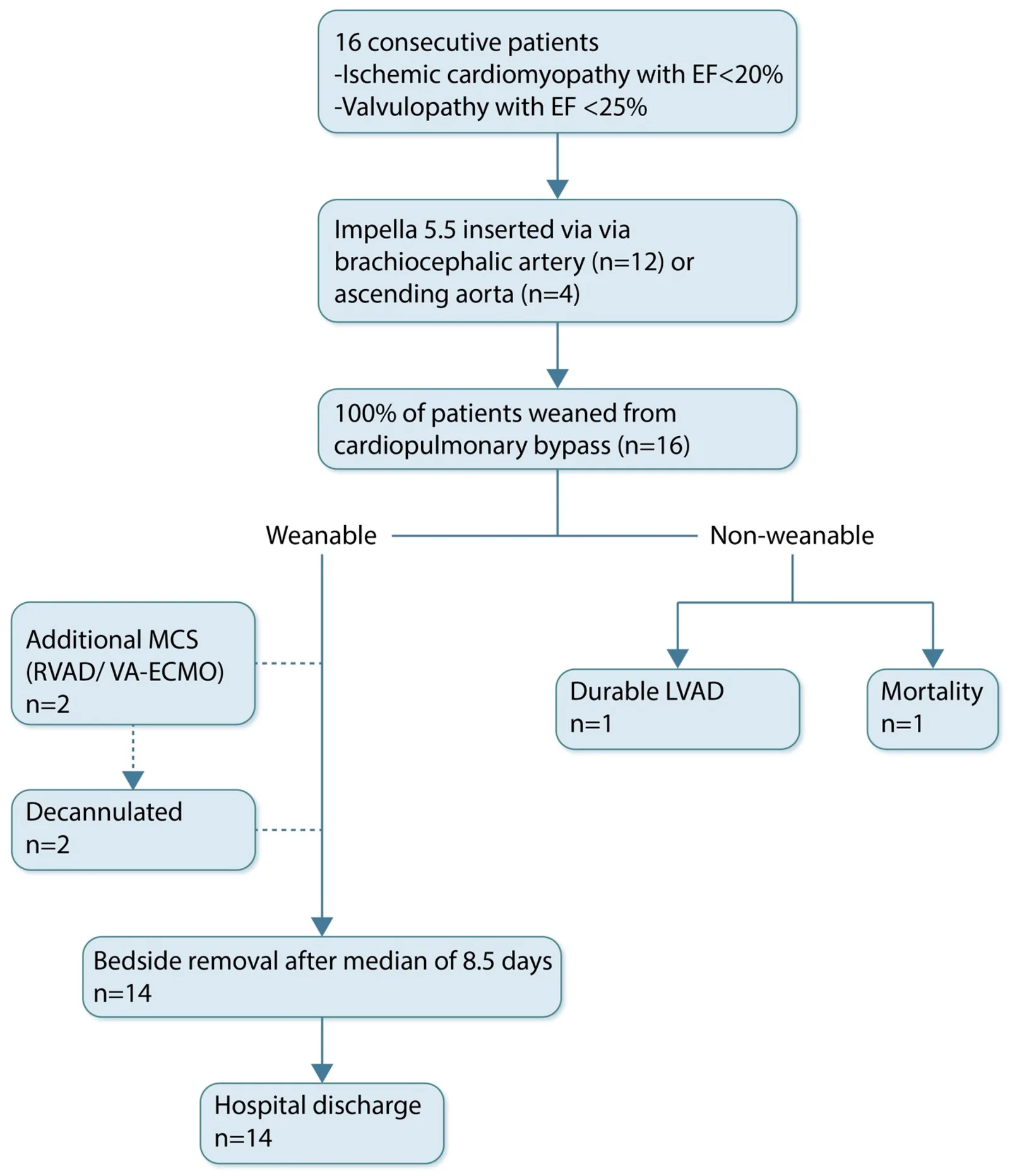
Flow chart of Impella 5.5-supported low-EF cardiac surgery including indications, postoperative course and outcomes. EF; ejection fraction, MCS; mechanical circulatory support, LVAD; left ventricular assist device, RVAD; right ventricular assist device, VA-ECMO; veno-arterial extracorporeal membrane oxygenation.

**Figure 2:**
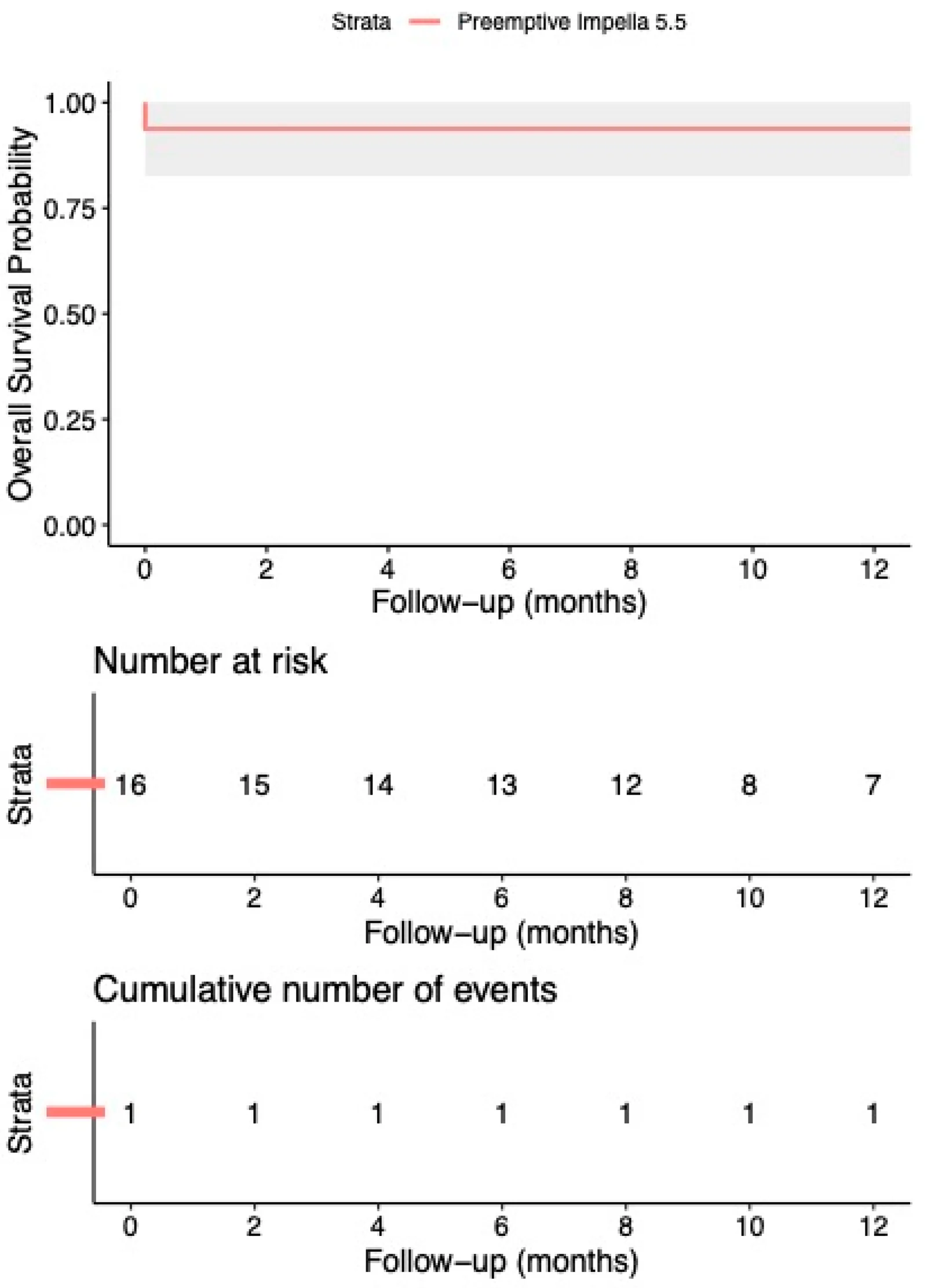
Kaplan–Meier survival. Freedom of all-cause mortality at 1 year was 95.2% with no further mortality over a median follow-up of 10 months (7.5 to 20.5 months).

**Table 1:** Baseline demographics and patient characteristics.

Variable	Periprocedural Impella 5.5 Support (*n* = 16)
Demographics	
Age, years	66.5 (52.5–70.0)
Male gender	15 (93.8)
STS-PROM, %	12.1 (5.3–14.2)
BMI (mg/m^2^)	24.8 (22.2–29.7)
HTN	9 (56.3)
HLD	8 (50.0)
AF	4 (25.0)
CAD	9 (56.3)
Prior PCI	4 (25.0)
DM	3 (18.8)
Renal failure	3 (18.8)
Dialysis-dependent	2 (12.5)
COPD	3 (18.8)
Smoker	7 (43.8)
Prior sternotomy	1 (6.3)
Preoperative cardiac function	
EF	20 (15.0–31.5)
LVEDD, mm	63.6 (53.5–64.5)
LVESD, mm	58.0 (40.5–60.0)

Data are presented as median (IQR), *n* (%). STS-PROM; Society of Thoracic Surgeons Predicted Risk of Mortality; BMI, Body Mass Index; HTN, hypertension; HLD, hyperlipidemia; AF, atrial fibrillation; CAD, coronary artery disease; DM, diabetes mellitus; COPD, chronic obstructive pulmonary disease; EF, ejection fraction; LVEDD; left ventricular end-diastolic diameter; LVESD, left ventricular end-systolic diameter.

**Table 2:** Intraoperative outcomes.

Variable	Periprocedural Impella 5.5 Support (*n* = 16)
Intraoperative characteristics	
Cardiac procedure performed	
Isolated CABG	6 (37.5)
Valvular procedures (AVR or MVR ± TVr)	4 (25.0)
Combined valvular procedures with CABG	6 (37.5)
Impella implant approach	
Brachiocephalic artery	12 (75.0)
Direct aortic	4 (25.0)
Additional mechanical support	
VA ECMO	1 (6.3)
Percutaneous RVAD	1 (6.3)
CBT, minutes	156.6 ± 52.3
ACT, minutes	108.9 ± 38.7

Data are presented as mean ± SD, *n* (%). CABG, coronary artery bypass graft; AVR, aortic valve replacement; MVR, mitral valve replacement; TVr, tricuspid valve repair; CBT, cardiopulmonary bypass time; ACT, aortic cross-clamp time; RVAD, right ventricular assist device.

**Table 3:** Postoperative characteristics.

Variable	Periprocedural Impella 5.5 Support (*n* = 16)
Postoperative outcomes	
Duration of support, days	8.5 (6.5–13.0)
ICU LOS, days	18.5 (11.0–32.5)
Hospital LOS, days	26.0 (17.0–63.5)
STS operative mortality	1 (6.3)
Overall mortality	2 (12.6)
CVA	
Non-disabling stroke, no residual deficits	2 (12.6)
De novo renal failure	2 (12.6)
Revision for bleeding	4 (25.2)
Discharge disposition	
Home	8/15 (53.3)
Rehab	6/15 (40.0)
Skilled nursing facility	1/15 (6.7)
Follow-up, months	10.0 (7.5–20.5)

Data are presented as median (IQR), *n* (%). ICU, intensive care unit; LOS, length of stay; STS, Society of Thoracic Surgeons operative mortality defined as all mortalities occurring during the index admission even if occurring after 30 days or all mortalities occurring after discharge from the index admission but within 30 days; CVA, cerebrovascular accident; non-disabling stroke was defined as a minor stroke without residual deficits at time of discharge; disabling stroke was defined as persistent deficits impairing daily activities.

## Data Availability

The raw data supporting the conclusions of this article will be made available by the authors on request.
